# Practice Variation in IgA Nephropathy: When the Field Evolves Faster Than Consensus

**DOI:** 10.34067/KID.0000001343

**Published:** 2026-08-27

**Authors:** George Vasquez-Rios, Luis Sanchez-Russo

**Affiliations:** 1 Glomerular and Genetic Diseases Center, Renal Medicine Associates, New Mexico; 2 Glomerular Disease Institute, Central Florida Kidney Specialists, Florida

**Keywords:** GN, IGA nephropathy, Mpgn (Membranoproliferative GN), Primary GN, FSGS

The management of IgA nephropathy (IgAN) has undergone remarkable transformations in contemporary nephrology. Over the past two decades, advances in disease biology, risk stratification, and therapeutic development have fundamentally reshaped previous clinical and therapeutic paradigms.^1^ Importantly, the implications—and the full extent—of these changes continue to challenge clinicians at present and should be carefully addressed when developing strategies aimed at promoting treatment consensus and evidence-based best practices.

In this issue of *Kidney360*, Barr *et al*.^2^ conducted a population-based retrospective cohort study evaluating physician-level variation in the management of IgAN in Manitoba, Canada, between 2002 and 2021, using registry data linked to administrative databases. They analyzed 380 patients with biopsy-proven IgAN managed by 38 nephrologists to quantify variation in two key aspects of care: the timing of kidney biopsy and glucocorticoid prescribing within 1 year after the index biopsy. Using hierarchical mixed-effects models, they found that the physician-level intraclass correlation coefficient for time to kidney biopsy was 0.31, indicating that approximately 31% of the overall variation in biopsy timing was attributable to between-physician clustering after adjustment for measured covariates. To facilitate clinical interpretation, the authors also calculated the median time ratio, which was 2.65, suggesting that two otherwise similar patients could experience nearly a three-fold difference in the time to kidney biopsy depending on the physician-level cluster in which they received care.

For glucocorticoid prescribing, the physician-level intraclass correlation coefficient was 0.07, indicating that approximately 7% of the variability in corticosteroid use was attributable to between-physician clustering after adjustment for measured covariates. The corresponding median odds ratio was 1.59, demonstrating that two otherwise comparable patients could have a 59% difference in the odds of receiving glucocorticoid therapy depending on the physician managing their care. In addition, higher levels of proteinuria were associated with shorter time to kidney biopsy, and this association was modified by rural residence, with the delay being most pronounced among rural patients with lower proteinuria. Higher proteinuria and the presence of crescents on kidney biopsy were associated with an increased likelihood of glucocorticoid prescription, whereas interstitial fibrosis and tubular atrophy involving more than 50% of the biopsy specimen was associated with a lower likelihood of treatment. Because physician identifiers were anonymized, potentially informative physician characteristics—such as years of clinical experience, fellowship training, subspecialty expertise, or practice style—could not be evaluated.

The interpretation of these findings requires careful consideration of the historical context in which these clinical decisions were made. Unlike diseases managed according to relatively stable therapeutic frameworks, IgAN has undergone profound changes that merit discussion. Consequently, much of what is identified statistically as physician-level variability may indirectly reflect the nuances of clinical decision making during a period characterized by rapidly evolving evidence, changing treatment recommendations, and substantial therapeutic uncertainty.

## Practice Variation Reflects More than Physician Preference

The study appropriately focuses on kidney biopsy timing and glucocorticoid prescribing because these represent clinically meaningful decisions that can be captured using administrative and pharmacy databases. Nevertheless, these variables may not be interpreted as isolated physician behaviors. Rather, they could reflect the cumulative influence of multiple factors that are difficult—or perhaps impossible—to fully measure in observational datasets, including the rapid evolution of evidence supporting immunomodulatory therapy in IgAN. During much of the study period (2002–2021), nephrologists practiced against a backdrop of conflicting clinical trial results, evolving interpretations of corticosteroid efficacy, and legitimate concerns regarding treatment-related toxicity. Successive studies—from early meta-analyses through Supportive Versus Immunosuppressive Therapy for the Treatment of Progressive IgA Nephropathy study, the initial Therapeutic Evaluation of Steroids in IgA Nephropathy Global trial, and ultimately the modified dose Therapeutic Evaluation of Steroids in IgA Nephropathy Global study published in 2022—fundamentally altered the perceived balance between therapeutic benefit and risk of using steroids in IgAN.^3–6^ These developments were subsequently reflected in successive iterations of the Kidney Disease Improving Global Outcomes (KDIGO) Glomerular Disease Guidelines, which progressively refined recommendations regarding patient selection and corticosteroid use.^7^ Consequently, physicians practicing in 2005, 2012, and 2021 were not making decisions on the basis of the same body of evidence.

## Practicing in an Era of Uncertainty

Similarly, considerable uncertainty surrounded the optimal timing of kidney biopsy throughout much of the study period. Until recently, there was no universally accepted framework defining which patients with isolated hematuria, low-grade proteinuria, or slowly progressive kidney dysfunction should undergo biopsy. Furthermore, the absence of highly effective disease-modifying therapies likely reinforced a more conservative diagnostic approach because the immediate therapeutic implications of establishing a tissue diagnosis were often perceived to be limited.

Clinical decision making was also influenced by factors that remain difficult to quantify. Physician perception of disease severity frequently extended beyond measured eGFR and proteinuria, incorporating subtle clinical features such as the trajectory of kidney function decline, anticipated treatment tolerance, and the overall clinical phenotype of the patient. Likewise, histopathologic interpretation was often influenced by individual weighting of chronicity versus activity, the significance attributed to crescents, or concern regarding irreversible fibrosis, which was acknowledged by Barr *et al*.^8^ These nuanced judgments—developed through clinical experience rather than formal algorithms—inevitably contributed to practice variation but cannot be fully captured through statistical adjustment.

Furthermore, as expected in this type of analysis, confounding by indication remains an important consideration, particularly when interpreting glucocorticoid prescribing. Although the authors adjusted for several clinically relevant variables, longitudinal measures of disease activity—such as the trajectory of kidney function decline—were not incorporated. This is particularly relevant because progressive loss of kidney function, even in the absence of rapidly progressive GN, may influence decisions regarding immunosuppressive therapy in clinical practice. These factors introduce residual confounding driven by clinical judgment and inevitably limit how confidently physician-level variability in corticosteroid prescribing can be interpreted.

## Landmark Clinical Trials, KDIGO Guideline Updates, and Adoption in Clinical Practice

As described previously, the management of IgAN evolved substantially throughout the study period, influenced by landmark trials, together with successive revisions of the KDIGO Clinical Practice Guidelines. Although the authors made significant efforts to address this issue by adjusting for era of diagnosis, broad categorical eras may incompletely account for the continuous evolution of clinical practice. Furthermore, adoption of emerging evidence is inherently heterogeneous as well. Consequently, part of the observed between-physician variation may reflect differences in the timing of evidence adoption rather than intrinsic differences in treatment philosophy. Modeling calendar time as a continuous variable—or incorporating physician-by-time interaction terms or random slopes for time—could potentially provide a more granular understanding of evolving practice patterns, although even these approaches would be unlikely to fully capture the complexity of clinical decision making in such a rapidly evolving therapeutic landscape.

**Figure 1:**
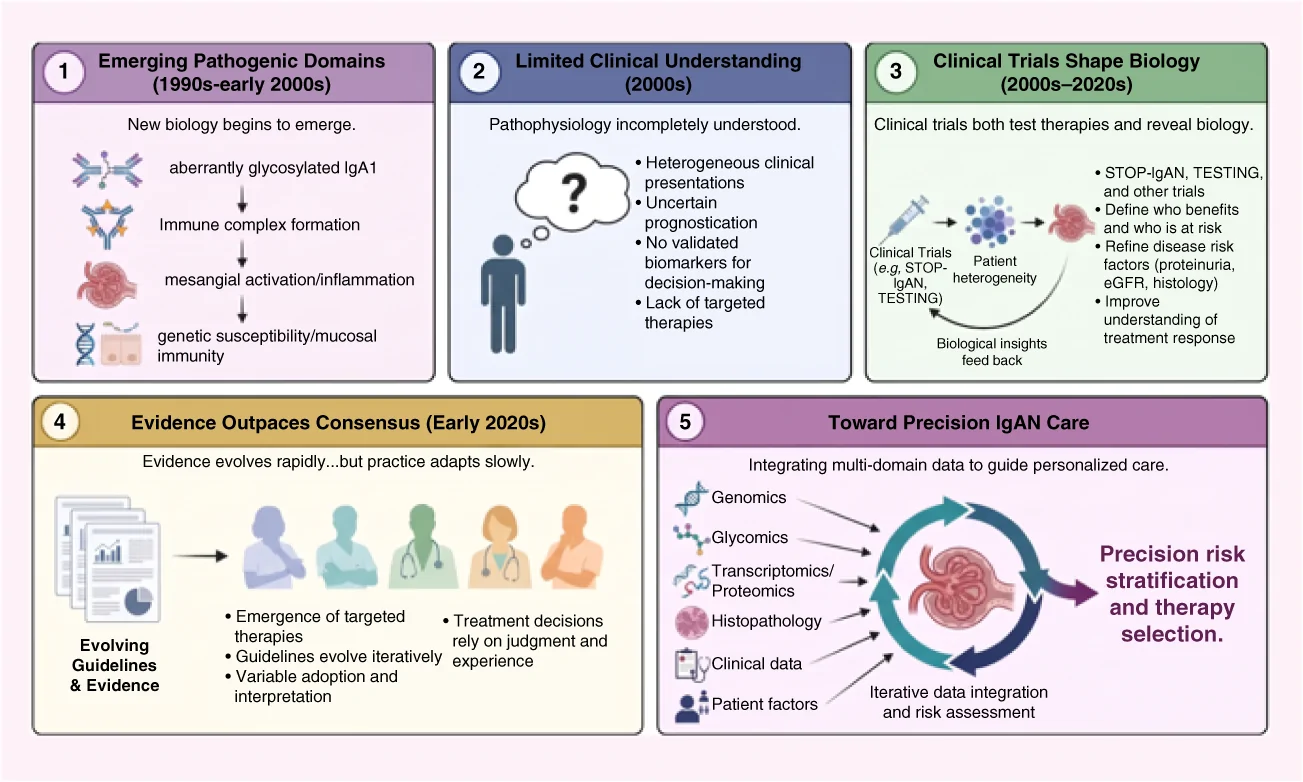
**The evolving landscape of IgAN pathobiology and clinical management.** (*1*) Discovery of aberrantly glycosylated IgA1 and its role in immune complex formation, mesangial activation, and genetic/mucosal immune susceptibility established the foundational IgAN disease model (1990s–early 2000s). (*2*) Incomplete understanding of pathophysiology, heterogeneous clinical presentations, uncertain prognostication, and a lack of validated biomarkers or targeted therapies (2000s).(*3*) Clinical trials (*e.g*., STOP-IgAN and TESTING) both tested therapies and generated biological insight, refining risk stratification (proteinuria, eGFR, and histology) in an iterative feedback loop based on clinical experience rather than clear algorithms, further led to clinical practice heterogeneity (2000s–2020s). (*4*) The rapid emergence of targeted therapies has outpaced consensus guidelines, which evolve iteratively amid variable adoption and clinician judgment (early 2020s and onward). (*5*) The field aims to move toward precision IgAN care, integrating multiomics, histopathology, clinical data, and patient factors for personalized risk stratification and therapy selection. IgAN, IgA nephropathy; STOP-IgAN, Supportive Versus Immunosuppressive Therapy for the Treatment of Progressive IgA Nephropathy study; TESTING, Therapeutic Evaluation of Steroids in IgA Nephropathy Global.

Taken together, these considerations raise a broader question: Are we truly measuring physician variability, or are we capturing the natural evolution—and challenging realities—of clinical practice in IgAN? A deeper question, therefore, may not be whether physicians practiced differently, but whether the field itself was evolving (and continues to evolve) faster than consensus could emerge, ultimately leading to practice pattern heterogeneity (Figure 1).

The work by Barr *et al*., represents an important methodologic advance for glomerular disease research and provides a valuable framework for understanding treatment patterns and the gap between guideline recommendations and their implementation in clinical practice. At the same time, their findings remind us that statistical models can quantify unexplained variation but may not fully distinguish variation arising from physician judgment, evolving scientific evidence, health system factors, referral pathways, or residual differences in patient complexity. Addressing these challenges, particularly in the contemporary era of IgAN management, will likely require a new generation of real-world evidence infrastructure capable of continuously evaluating treatment effectiveness, net clinical benefit, and patient outcomes while integrating longitudinal clinical, pathologic, and emerging molecular data to better inform therapeutic decision making.^9^ Accordingly, we suggest that the observed variability may be more cautiously interpreted as practice-pattern variation; a broader construct encompassing physician judgment, evolving scientific evidence, structural health care factors, and the therapeutic uncertainty that has characterized modern IgAN management.

## Supplementary Material
