# Supplementary material for: Practice Variation in IgA Nephropathy: When the Field Evolves Faster Than Consensus
Source: Kidney360. 2026 Aug 27;7(8):1697–9. doi: 10.34067/KID.0000001343 (PMC13567841; doi:10.34067/KID.0000001343)
Supplement: Supplementary file 1 [file kidney360-7-1697-s001.pdf]

ASN American Society of Nephrology
JASN Journal of the American Society of Nephrology
KIDNEY360 Accessing Our World From Every Angle
CJASN Clinical Journal of the American Society of Nephrology

ASN Journal Disclosure Form

As per ASN journal policy, I have disclosed any financial relationships or commitments I have held in the past 36 months as included below. I have listed my Current Employer below to indicate there is a relationship requiring disclosure. If no relationship exists, my Current Employer is not listed.

L. Sanchez Russo reports the following:
Employer: Central Florida Kidney Specialists; Consultancy: ANI Therapeutics: Consultant ; Vertex Pharmaceuticals: Consultant ; Vera Therapeutics: Consultant ; Apellis: Consultant ; Travere: Advisory Board; Calliditas Therapeutics (Advisory Board); Honoraria: ANI Pharmaceuticals (Speaker Bureau); Otsuka (Speaker Bureau); Vertex (Speaker Bureau); Vera (Speaker Bureau); Advisory or Leadership Role: Calliditas Therapeutics (Advisory Board); ANI Pharmaceuticals (Advisory Board), Travere (Advisory Board); and Speakers Bureau: ANI Pharmaceuticals (Speaker Bureau); Otsuka (Speaker Bureau); Vertex (Speaker Bureau); Vera (Speaker Bureau); Travere (Speaker Bureau).

I understand that the information above will be published within the journal article, if accepted, and that failure to comply and/or to accurately and completely report the potential financial conflicts of interest could lead to the following: 1) Prior to publication, article rejection, or 2) Post-publication, sanctions ranging from, but not limited to, issuing a correction, reporting the inaccurate information to the authors' institution, banning authors from submitting work to ASN journals for varying lengths of time, and/or retraction of the published work.

Name: Luis F. Sanchez Russo
Manuscript ID: K360-2026-000883
Manuscript Title: Practice Variation in IgA Nephropathy: When the Field Evolves Faster Than Consensus
Date of Completion: July 23, 2026
Disclosure Updated Date: July 23, 2026

ASN American Society of Nephrology | JASN Journal of the American Society of Nephrology | KIDNEY360 Accessing Our World From Every Angle | CJASN Clinical Journal of the American Society of Nephrology

ASN Journal Disclosure Form

As per ASN journal policy, I have disclosed any financial relationships or commitments I have held in the past 36 months as included below. I have listed my Current Employer below to indicate there is a relationship requiring disclosure. If no relationship exists, my Current Employer is not listed.

G. Vasquez-Rios reports the following:
Employer: Renal Medicine Associates; Consultancy: Yes. I have received consulting compensation from pharmaceutical and biotechnology companies, including Vertex Pharmaceuticals, Vera Therapeutics, Otsuka Holdings, Natera, Amgen, Apellis, Novartis, and Travere Therapeutics.; Ownership Interest: Yes. I hold individual stocks in Vertex Pharmaceuticals, Vera Therapeutics, and Otsuka Holdings. No other ownership interests to disclose.; Research Funding: Yes. I have served as a principal or named investigator on research projects funded, in whole or in part, by for-profit companies, including Otsuka, Calliditas Therapeutics, and Natera. In accordance with disclosure guidelines, this includes research funding provided to my institution and managed on my behalf.; Honoraria: Yes. I have received honoraria from pharmaceutical and biotechnology companies within the past 24 months, excluding certified continuing education activities, including Vertex Pharmaceuticals, Vera Therapeutics, Otsuka Holdings, and Calliditas Therapeutics.; Advisory or Leadership Role: Yes. I have served in compensated advisory roles for pharmaceutical and biotechnology companies within the past 24 months for the companies described above. I have not held officer or board member positions in for-profit companies.; and Speakers Bureau: Yes. I have participated on speaking rosters for for-profit companies within the past 24 months, including Natera, Vertex Pharmaceuticals, Vera Therapeutics, Otsuka Holdings, and Calliditas Therapeutics.

I understand that the information above will be published within the journal article, if accepted, and that failure to comply and/or to accurately and completely report the potential financial conflicts of interest could lead to the following: 1) Prior to publication, article rejection, or 2) Post-publication, sanctions ranging from, but not limited to, issuing a correction, reporting the inaccurate information to the authors' institution, banning authors from submitting work to ASN journals for varying lengths of time, and/or retraction of the published work.

Name: George Vasquez-Rios
Manuscript ID: K360-2026-000883
Manuscript Title: Practice Variation in IgA Nephropathy: When the Field Evolves Faster Than Consensus
Date of Completion: July 23, 2026
Disclosure Updated Date: July 23, 2026
